# Preventive Transhepatic Tract Embolisation after Percutaneous Biliary Interventions: A Systematic Review

**DOI:** 10.1155/2020/8849284

**Published:** 2020-10-05

**Authors:** Daniel Schmitz, De-Hua Chang, Jochen Rudi, Svetlana Hetjens, Matthias P. A. Ebert

**Affiliations:** ^1^Department of Gastroenterology, Oncology and Diabetology, Theresienkrankenhaus und St. Hedwigsklinik GmbH, Heidelberg University, Bassermannstr.1, 68165 Mannheim, Germany; ^2^Department of Diagnostic and Interventional Radiology, University Hospital of Heidelberg, Im Neuenheimer Feld 110, 69120 Heidelberg, Germany; ^3^Department of Medical Statistics, Biomathematics and Information Processing, Mannheim University Hospital, Ludolf-Krehl-Str. 13-17, 68165 Mannheim, Germany; ^4^Department of Medicine II, Medical Faculty Mannheim, Heidelberg University, Theodor-Kutzer-Ufer 1-3, 68167 Mannheim, Germany

## Abstract

Preventive transhepatic tract embolisation (PTTE) after percutaneous biliary intervention (PBI) may reduce adverse events. The aim of this systematic review was to analyse feasibility, safety, and efficacy of PTTE with different embolic agents. A systematic literature research was performed according to the PRISMA guidelines. The identified studies were analysed concerning study quality, number of cases, indication, embolic agent, embolisation technique, success, and embolisation-related adverse events. Out of 62 identified records, 7 studies of mainly moderate study quality published through 2019 were included for further analysis. Cyanoacrylate (*n* = 4), gelatin sponge (*n* = 2), and coils (*n* = 1) were used as embolic agents in a total number of 314 patients. Technical success was 96–100%. Embolisation-related adverse events (glue migration, pain) occurred in 10/314 (3.2%) patients. Reduction of PBI-related pain was approved by one controlled study; haemorrhage events were reduced but not clearly significant. Overall, biliary leak, transhepatic bleeding, and PBI-related pain occurred in 7/201 (3.5%), 1/293 (0.3%), and 17/46 (36.9%) documented patients after PTTE. Adverse events which likely could not have been prevented by PTTE occurred in 23/180 (12.8%) patients. Embolic agents were not compared. In conclusion, PTTE is feasible and safe. It is effective concerning the prevention of PBI-related pain, and it may be effective concerning haemorrhage. Prevention of biliary leak is not proven. It remains unclear which embolic agent should be preferred. A prospective randomised trial including all preventable adverse events is lacking.

## 1. Introduction

**Figure 1:**
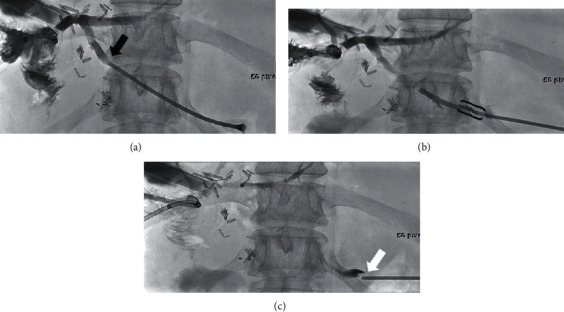
Case example with fluoroscopy images of a patient with hemihepatectomy for cholangiocellular carcinoma who received PTTE with gelatin foam after percutaneous transhepatic balloon dilation of a stenosis of the hepaticojejunal anastomosis (Department of Diagnostic and Interventional Radiology of University Hospital of Heidelberg). (a) The 6 F-sheath is withdrawn from a dilated biliary duct (black arrow) by continuous injection of a contrast agent. (b) Injection of gelatin foam through a side port of the sheath into the transhepatic tract (black brackets). (c) Detachment of gelfoam (white arrow) from the sheath tip marks the liver capsule.

Percutaneous biliary intervention (PBI) can be associated with high rates of different adverse events [1, 2], which can be reduced by the use of an ultrasound-guided percutaneous bile duct access [3], stent insertion in a single session without remaining external catheter [4], or left-sided bile duct access [5]. Preventive transhepatic tract embolisation (PTTE) (Figure 1) might be a further effective measure to reduce adverse events after PBI. It was first applied in patients with high bleeding risk after liver biopsy [6]. Hereafter, it was introduced in patients after PBI [7]. However, it is not well known which adverse events can be prevented by transhepatic tract embolisation, which embolic agent should be preferred, and whether PTTE itself is related to adverse events. Endoscopic ultrasound-guided biliary drainage/interventions (EUS-BD) are increasingly used for similar indications as PBI. A recently published meta-analysis has concluded that EUS-BD may be preferred over percutaneous transhepatic biliary drainage (PTBD) as EUS-BD is associated with significantly better clinical success and a lower rate of adverse events [8]. Therefore, adverse event rates of EUS-BD may be used as a reference point for improved performance of PBI. The aim of this systematic review was to analyse all published studies on PTTE regarding feasibility, safety, and probable efficacy of the used embolic agents.

## 2. Materials and Methods

This systematic review was performed in accordance with the PRISMA guidelines [9].

### 2.1. Data Sources and Search Strategy

An experienced medical librarian (V. B.) developed the search strategy and performed the literature search. PubMed, Embase, and Cochrane database were searched using a search strategy developed to identify all papers on embolisation in association with PBI regardless of study design. The following search terms were used: “Percutaneous Biliary Intervention,” “Embolization, Therapeutic,” “Embolotherapy,” “Embolization,” “Closure” and “Liver,” “Liver Tract,” “Transhepatic Tract,” “Bile Duct,” “Biliary Duct.” The search included all articles published through 16 May 2019. All results were downloaded into EndNote X9 (Clarivate Analytics, Boston, USA), a bibliographic database manager. To further increase our possible search results, we manually searched articles through the references of the retrieved publications. Based on the title and abstract, we selected articles for full-text review. Duplicate publications and articles not published in English or German were removed.

### 2.2. Inclusion and Exclusion Criteria

The following criteria were defined as inclusion criteria: comparative and noncomparative studies concerning preventive liver tract embolisation after PBI using any kind of embolic agent should be included. Case reports or case series less than 3 cases on preventive transhepatic tract embolisation as well as studies on percutaneous therapeutic bile duct embolisation in biliary leak should be excluded as the aim of this study was preventive embolisation. Studies had to report at least 1 of the following: technical success which was defined as successful closure of the transhepatic tract proven by injected contrast medium during fluoroscopy; clinical success which was defined as a reduction of PBI-associated adverse events in comparison with a control group without preventive transhepatic tract embolisation, or embolisation-related adverse events.

### 2.3. Study Selection and Data Extraction

Two reviewers (DS, DC) independently assessed the eligibility and validity of each study as well as the extracted data. Extracted data included study design, year of the study, number of cases, indications for PBI, the embolic agent used, the technique of embolisation, technical as well as clinical success of embolisation, PBI-associated adverse events, and embolisation-related adverse events. Embolisation-related adverse events were retrospectively classified according to the CIRSE classification system of complications in interventional radiology [10]. Accordingly, six grades of complications were differentiated: Grade 1: complication during the procedure, which could be solved within the same session; Grade 2: prolonged hospital stay (<48 h); Grade 3: additional postprocedure therapy or prolonged hospital stay (>48 h) required; Grade 4: permanent mild sequelae; Grade 5: permanent severe sequelae; and Grade 6: death.

### 2.4. Study Quality Assessment

For study quality assessment, we used the Newcastle-Ottawa scale (NOS) [11]. The NOS measures quality in the 3 parameters of selection, comparability, and outcome and awards a maximum of 4, 2, and 3 stars, respectively. High-quality studies score over 7 on this scale, moderate quality studies score between 5 and 7, and low-quality studies score under 5. Two authors (DS and DC) evaluated the quality of studies independently, with any disagreement between them to be discussed with a third reviewer (SH) and agreement reached by consensus.

## 3. Results

### 3.1. Search Strategy Yield and Quality Assessment

**Figure 2:**
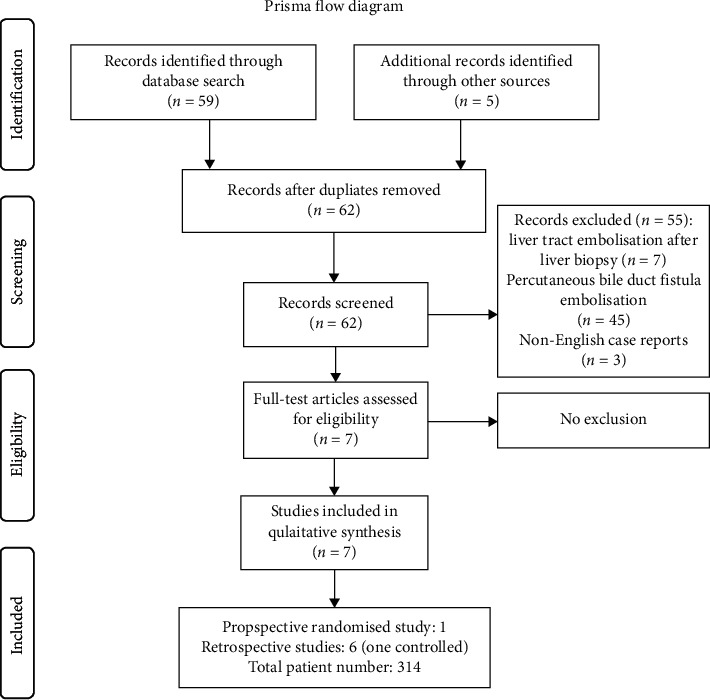
Screening and inclusion process are shown in a flow diagram according to the PRISMA statement (http://www.prisma-statement.org).

Fifty-nine records were identified through database searching, and 5 additional records were identified through other sources. After having removed all duplicated records, all 62 search results were screened. Fifty-five records concerning liver tract embolisation after liver biopsy (*n* = 7), percutaneous bile duct fistula embolisation (*n* = 45), and non-English case reports (*n* = 3) were excluded. Seven published full-text articles were assessed for eligibility. As there were no case reports, all seven studies [7, 12–17] were included in qualitative synthesis: 1 prospective randomised study, 1 controlled before-after study, and 5 uncontrolled retrospective studies. All studies were published in the period from 2000 to 2019. The search strategy is summarized in a PRISMA flow diagram (Figure 2).

All 7 studies were single center based. A total of 314 patients with embolisation were included in the analysis. Meta-analysis was not performed since only two studies had controls without embolisation comprising a further 123 patients.

Based on the Newcastle-Ottawa scale assessment, 1 study was of high quality [15], 5 were of moderate quality [7, 12–14, 16], and 1 was of low quality [17]. Quality assessment is summarized in Table 1.

### 3.2. Analysis

The indication for PBI was mainly malign bile duct obstruction (not always specified) in all studies with 252/314 (80.3%, range: 61.9–100%). Patients with malign bile duct obstruction were mixed with patients with an underlying benign disease such as bile duct stones in 5 of 7 studies [7, 12, 14–16]. Only in two retrospective nonrandomised cohort studies, all patients showed a malign bile duct obstruction with concomitant ascites [13, 17].

Cyanoacrylate followed by *N*-butyl cyanoacrylate (NBCA) was used as an embolic agent in 4 studies [7, 12–14], gelatin in 2 [15, 16], and coils in 1 study [17].

Overall, the technical success of embolisation was very high in all studies with 99.0 (96.0–100%).

Adverse events related to embolisation were rare (10/314 (3.2%)) and were exclusively observed in two patients with glue migration after cyanoacrylate embolisation. In one patient, a small amount of fragmented glue was detected by a CT scan outside the biliary stent but did not cause any symptoms [14]. In the other patient, the occluded biliary metal stent could be reopened by an inserted balloon angioplasty catheter [13]. The pain was related to cyanoacrylate injection in one study (8/42 (19.0%)) and qualified as mild (visual analogue scale ≤5) [16]. However, this was the only study in which the patients received local anaesthetics and did not receive analgosedation.

In the two studies with patients with malign ascites, embolisation was combined with the insertion of a self-expandable metal stent as a one step-procedure [13, 17]. The detailed techniques of transhepatic tract embolisation are summarized in Table 2.

Clinical efficacy of PTTE could be assessed in two studies with controls without embolisation [12, 15]. The first study with cyanoacrylate embolisation which exclusively reported the effect of embolisation on PBI-related pain was the only study that used a visual analogue score (VAS) and a required analgesic score (RAS) for pain quantification [12]. The pain reported by the patient was shown to be significantly lower in the embolisation group, compared with the nonembolisation group (*p* < 0.0023 and *p* < 0.002, respectively). The second study with gelatin embolisation which exclusively reported the effect of embolisation on PBI-related haemorrhage showed a reduction of bleeding events and lesser blood transfusions but failed to show a significant difference to the nonembolisation group if only bleeding events proven by imaging methods were calculated (6/101 versus 1/92; *p*=0.074) [15]. In the 5 cohort studies without a control group in which efficacy of PTTE could not be calculated, adverse events related to PBI were reported in follow-up periods with a median from 66 to 361 days [7, 13, 14, 16, 17]. Overall, adverse events related to PBI which should have been prevented by PTTE such as a biliary leak, transhepatic tract bleeding, and pain were observed in 7/201 (3.5%), 1/293 (0.3%), and 17/46 (36.9%) patients (Table 3).

Furthermore, adverse events that probably could not have been prevented by PTTE such as cholangitis, 2 tract metastasis after 30 days and 12 months, arterial intrahepatic haemorrhage, haemobilia, and nonbiliary pleural effusion were observed in 23/180 (12.8%) patients. As mentioned above, PBI-related pain was quantified in only two studies [12, 13].

## 4. Discussion

Preventive liver tract embolisation after PBIs was technically successful in almost all patients, and embolisation-related adverse events were rare. These review results suggest that PTTE is feasible and safe. However, the efficacy of PTTE cannot be clearly assessed on the basis of the present seven studies as only two of them had a control group without embolisation.

### 4.1. Pain

The only one prospective randomised study with a short follow-up of a few days using cyanoacrylate as an embolic agent showed a significant reduction of PBI-related pain. It did not report in which group other important adverse events as haemorrhage or biliary leak occurred [12]. Postprocedural pain is observed after PBI in up to one-third of the cases [18]. It may derive from peritoneal irritation by intraperitoneal bleeding or biliary leak, liver capsule injury, dilation of the transhepatic tract, or bile duct stenosis, or from remaining external drainage especially if right-sided intercostal liver access was chosen [5, 19]. Theoretically, PTTE is most likely effective in the prevention of pain that derives from peritoneal irritation by intraperitoneal bleeding or biliary leak. However, the intriguing reduction of pain in this study remains not fully understood.

### 4.2. Haemorrhage

The second controlled study, which was a retrospective before-after study focussing on haemorrhage as an adverse event, showed a significant reduction of bleeding complications from 12.0% to 3.0% when the number of blood transfusions was combined with the number of visualised bleeding events by imaging methods [15]. Even if the study failed to show a significance in the reduction of visualised bleeding events, the incidence of 1.9% bleeding events is relatively low if compared with 14.7% (including haemobilia) bleeding events which had been reported in one of the rare prospective studies on PBI without PTTE [18]. The portal vein, the hepatic vein, or the hepatic artery vessels may by injured by PBI. Consecutively, bleeding may occur as bleeding into the bile duct (haemobilia) manifesting as gastrointestinal bleeding or bleeding through the biliary drainage catheter, bleeding into the liver parenchyma, bleeding into the liver capsule, or bleeding through the transhepatic tract into the abdominal or pleural cavity [19]. Theoretically, PTTE is likely most effective in the prevention of venous transhepatic tract bleeding and less likely effective in large vessel arterial bleeding and some kind of haemobilia if vessel injury is close to the bile duct.

### 4.3. Biliary Leak

An effect of PTTE on biliary leak was not proven by any of the 7 studies as it was not tested, or negative control was lacking. Therefore, the true effect of PTTE on biliary leak still needs to be proven. However, the cumulative incidence of biliary leaks was quite low with 7/201 (3.5%) cases. Comparison with historical study data concerning biliary leak is difficult as multiple variables may influence the incidence of the biliary leak in PBIs such as by a remaining external catheter. However, a recent meta-analysis on PBI versus EUS-guided biliary drainage found an incidence of 30/151 (19.8%) biliary leaks in PBI without PTTE [8].

### 4.4. Other Adverse Events

Adverse events that might be not preventable by transhepatic tract embolisation such as cholangitis or biliary sepsis, arterial intrahepatic haemorrhage, nonbiliary pleural effusion, or tract metastasis occurred in 23/180 (12.8%) of documented patients. These adverse events show the possible limitation of PTTE on the one side and the necessity of an adequate follow-up period on the other side as two of the tract metastases were observed several months after PBI.

### 4.5. Cyanoacrylate

Cyanoacrylate is successfully used in interventional radiology and gastrointestinal endoscopy for a long time and dedicated applications are commonly available. However, glue migration as an adverse event was reported in two studies. In one patient, the occluded biliary metal stent had to be reopened by the insertion of an angioplasty balloon catheter. Although cyanoacrylate-related adverse events were rare and without relevant severe consequences, it has to be kept in mind that cyanoacrylate glue deposition is inherently not always predictable, and nontarget embolisation, venous migration, microcatheter blockage, and catheter retention might occur [20, 21].

### 4.6. Gelatin Sponge

Manually prepared gelatin torpedoes or gelatin pledgets approved for liver tract sealing after liver biopsy were applied in two retrospective, uncontrolled cohort studies [15, 16]. No embolisation-related adverse event was observed. In contrast to permanent embolic agents, gelatin sponge is usually absorbed within 4–6 weeks although the occlusion persists due to an inflammatory reaction [22]. However, it would be beneficial if an approved medical device would be available for the application of gelatin sponge pledgets in different diameters and lengths. A further promising resorbable embolic agent might be microfibrillar collagen as one retrospective study on percutaneous portal vein intervention had shown significantly fewer bleeding events after preventive tract embolisation with microfibrillar collagen paste in comparison with gelatin [23].

### 4.7. Coils

The one available study on PTTE with coils showed no bile leak and no transhepatic tract bleeding in 16 patients with malign ascites although bile fluid or bleeding was not routinely ruled out by paracentesis [17]. The principle of vascular coil embolisation is vascular occlusion with subsequent vascular thrombosis and may work in the transhepatic tract concerning bleeding but may be questionable concerning bile fluid. Therefore, coils were combined with gelatin or cyanoacrylate for embolisation of large bile duct fistula in some case reports that were not included in this analysis [24–26]. Furthermore, it must be kept in mind that coil migration may occur months later after embolisation [27].

### 4.8. Limitations and Future Perspective

The significance of this review is weakened by the few available studies (*n* = 7), the lack of randomised studies (only one included), and some inaccurate outcome definitions (which kind of haemorrhage can be prevented). Therefore, it is not clear whether so much effort should be put into commonly used PTTE [28] although pain can be effectively prevented by PTTE, haemorrhage might be prevented by PTTE, and the overall biliary leak was not frequently (3.5%) observed after PTTE.

A prospective, randomised (multicentre) study should be performed that examines all three likely relevant adverse events such as haemorrhage, biliary leak, and pain (quantified pain score). This study should have an adequate case number to show a significant impact of PTTE on PBI-related adverse events. Keeping in mind reported incidences of haemorrhage (without haemobilia) and biliary leak in PBI with PTTE in this review of 0.3% and 3.5%, respectively, compared with the reported data on PBI without PTTE of 3.3% and 19.8%, respectively, in the abovementioned meta-analysis [8], a case number of 339 for haemorrhage or 66 for biliary leak should be achieved each in the embolisation and the nonembolisation groups according to a Chi-Square test simulation if *α* = 5%, a test power of 80%, and 10% dropouts are chosen.

## 5. Conclusions

PTTE is feasible and safe. It is effective concerning the prevention of PBI-related pain, and it may be effective concerning haemorrhage. The prevention of biliary leak is not proven. It remains unclear which embolic agent should be preferred. A prospective randomised trial including all preventable adverse events is lacking.

## Figures and Tables

**Table 1:** Summary of critical appraisals of included studies using the Newcastle-Ottawa quality assessment scale.

		Selection				Comparability		Outcome			Study quality (number of stars)
Embolic agent	Study	Representativeness of the intervention group	Selection of nonintervention group	Documentation of intervention	Outcome is not present at the beginning of the study	Most important endpoint	Additional endpoints	Assessment of outcome	Follow-up long enough	Follow-up complete	
Cyanoacrylate	Schmitz-R 2000	^*∗*^		^*∗*^	^*∗*^			^*∗*^	^*∗*^	^*∗*^	Moderate (6)
	Lyon 2006	^*∗*^	^*∗*^	^*∗*^	^*∗*^		^*∗*^	^*∗*^	^*∗*^		Moderate (7)
	Seif 2013	^*∗*^		^*∗*^	^*∗*^			^*∗*^	^*∗*^	^*∗*^	Moderate (6)
	Hwang 2019	^*∗*^		^*∗*^	^*∗*^			^*∗*^	^*∗*^		Moderate (5)
Gelatin	Dale 2015	^*∗*^	^*∗*^	^*∗*^	^*∗*^	^*∗*^		^*∗*^	^*∗*^	^*∗*^	High (8)
	Augustin 2019	^*∗*^		^*∗*^	^*∗*^			^*∗*^	^*∗*^		Moderate (5)
Coils	Sofue 2012	^*∗*^		^*∗*^					^*∗*^	^*∗*^	Low (4)

**Table 2:** Techniques of preventive transhepatic tract embolisation, technical success, and embolisation-related adverse events graded according to the CIRSE classification.

Embolic agent	Author/year	Technique of transhepatic tract embolisation	Technical success (%)	Embolisation-related adverse events (%)
Cyanoacrylate	Schmitz-R 2000	NBCA and iodised oil (50 : 50); injected through a 3F-PTFE-catheter; 7F-or 9F-port	20/20 (100.0%)	0/20 (0.0%)
	Lyon 2006	NBCA and iodised oil (50 : 50); injected through an 8F-dilator catheter	21/21 (100.0%)	0/20 (0.0%)
	Seif 2013	NBCA and iodised oil (80 : 20); injected through a 6F-dilator catheter	24/25 (96.0%)	Glue migration: CIRSE 3°: 1/25 (4.0%)
	Hwang 2019	NBCA/iodised oil (50 : 50; 40 : 60; 33 : 66), autologous blood, injected through an 8/14F-dilator catheter	41/42 (97.6%)	1/42 (2.4%) glue migration: CIRSE 1°, pain: 8/42 (19.0%)
Gelatin	Dale 2015	Gelatin foam pledgets, 14G/2 cm length, 2-3, one with radiopaque marker, push rod stylet, 8F-port	92/92 (100.0%)	0/92 (0.0%)
	Augustin 2019	Gelatin sponge torpedoes, manually prepared, delivered by pushing catheter/flushing, 8/11F-port	97/98 (98.9%)	0/98 (0.0%)
Coils	Sofue 2012	Metallic coils: 1–3 (5 mm × 5 cm; 4 mm × 3 cm; 3 mm × 4 cm); 6.5F-port	16/16 (100.0%)	0/16 (0.0%)
All			311/314 (99.0%)	10/314 (3.2%)

**Table 3:** PBI-related adverse events after percutaneous biliary intervention, preventable by transhepatic tract embolisation or not. The follow-up period in days (median).

Embolic agent	Author/year	Follow-up (days)	Biliary leak	Liver tract bleeding	PBI-related pain	Adverse events not preventable by tract embolisation
Cyanoacrylate	Schmitz-R 2000	95	1/20 (5.0%)	0/20 (0.0%)	Not tested	1/20 (5.0%): tract metastasis after 30 days
	Lyon 2006	1	Not reported	Not reported	6/21 (28.6%)	3/21 (14.3%): biliary sepsis (2), haemobilia (1)
	Seif 2013	182	2/25 (8.0%)	0/25 (0.0%)	11/25 (44.0%)	4/25 (16.0%): cholangitis
	Hwang 2019	58	1/42 (2.4%)	0/42 (0.0%)	Not tested	Not reported
Gelatin	Dale 2015	10	Not reported	1/92 (1.9%)	Not tested	Not reported
	Augustin 2019	361	3/98 (3.1%)	0/98 (0.0%)	Not tested	7/98 (7.1%): cholangitis (5), arterial haemorrhage (1), tract metastasis (1) after 12 months
Coils	Sofue 2012	66	0/16 (0.0%)	0/16 (0.0%)	Not tested	8/16 (50.0%): pleural effusion (4), cholangitis (2), haemobilia (2)
All			7/201 (3.5%)	1/293 (0.3%)	17/46 (36.9%)	23/180 (12.8%)

## Data Availability

All available data are included in the manuscript.
